# Modern Sociology

**Published:** 1900-11-10

**Authors:** 


### Modern Sociology.

### DANGEROUS TRADES.

### II.—Grindstones.

It will easily be believed that the workmen who have to do with grinding will suffer from those dust diseases of which we have already spoken. This is true, but they are liable to other accidents, which, as it seems, might be avoided. The great danger in working with a grindstone is that it may "fly," that is, break suddenly while in use, in which case the fragments would be scattered about with immense force, and anyone who was hit by one would suffer serious injury.

Grinding is a necessary process of the manufucture of steel tools of all sorts, from a needle to a sword-blade, and many large surfaces, from those of stoves to the armour-plating of our battle-ships, have also at some part of their manufacture to undergo a grinding process. The stones vary in size according to the work they have to do. The speed at which they are turned also varies, and while some are used wet, it is necessary for the grinding of some things, such as forks, augers, hammers, needles, and razors, that the stones should be dry. The velocity at which the stones are run in needle grinding is terrible. The Commissioners found that at Redditch, the centre of the needle industry, it was common for a stone, 30 inches in diameter, to perform 1,500 revolutions a minute. This gives a surface velocity of 11,700 feet per minute. Large stones, 6 feet in diameter, attain 200 revolutions per minute, and a surface velocity of 3,700 feet per minute. The force with which a fragment of either of these stones would strike any object can be imagined.

A stone which "flies" is a stone that is faulty. The faintest flaw or crack in the stone may be the cause of a fatal accident. Therefore, the first precaution to be taken is to see that the stones are sound. The responsibility thus rests, in the first place, with the owners of quarries, that they do not sell stones in which there is any flaw which they might discover. To this end the Commissioners have made several recommendations. First, that the quarrymen in quarries where grindstones are made should not be paid by piecework, as at present. If a man knows that he will not be paid for work spent on a faulty stone he is tempted to conceal flaws or defects in the stone. Secondly, the Commissioners advise that explosives should not be used for blasting in quarries where grindstones to be run by power are made. The shock which the stone sustains by blasting often causes cracks in the stone, which are not apparent at once, but which are liable to cause fracture when it is being run at a high velocity. Soft stones also are unsuitable for wheels which are to be run at a high speed, though they may be serviceable enough for those that are to be run by hand. Again, a flaw which is not large or serious enough to affect the safety of the stone at first may become serious after it has been used for some time. This may happen through two causes. The increased rate of speed at which it is necessary to run the smaller stone in order to get the same surface velocity as it had when it was larger may affect its solidity, and this may occur equally through the greater relative importance which the flaw has to the remaining mass of the stone. Stones which have been re-dressed are particularly liable to fracture. Occasionally a stone is returned to the quarry as being faulty, but the quarry-owner, if unscrupulous, may redress it, so as to conceal the flaw, and sell it as sound. Or even if the original flaw be got rid of, another may be developed in the further dressing. It is impossible to forbid the selling of such stones, for a stone which will not stand the strain of a high rate of speed may be perfectly suitable for work which does not demand great velocity. Moreover, grinders will sometimes buy a faulty stone because it is cheap, and take the chance of its flying.

There is a considerable liability to flying among stones used for wet grinding. If these are left, when not in use, resting in the water, the lower side of the stone becomes more or less saturated with water, and develops what the grinders call a "heavy side." When a stone is running with one side heavier than the other, there is great danger of its flying. Careless storing of stones may also lead to a heavy side. A stone left unprotected in a yard may get wet, and the rain will naturally percolate down until the lower side is much heavier than the other. To the danger of heavy side with wet grinding must be added the risk of fracture after the wet stone has been subjected to the action of frost. "It is an undoubted fact," says the report, "that during every severe frost at Sheffield there occurs an epidemic of grindstone accidents." Among the witnesses examined, there was found to be great difference of opinion as to whether it was better to protect grindstones from the weather or to expose them to it, some holding to the notion that when a stone has been "weathered," it gets hardened and better compacted by its exposure to cold, heat, and damp, and that flaws which would remain hidden until the stone "flies" are thus brought to light; but, on the other hand, the flaws may be created by these very conditions, and a stone spoiled which otherwise might be serviceable.

Sometimes the stones are hung in the "trow" or "stone hole" where they are used by means of wedges, and sometimes by iron plates. The Commissioners came to the conclusion that the wedges were unsafe, as they were sometimes forced in too tightly, and sometimes swelled through the action of damp after they had been inserted, in either case tending to produce cleavage. When wedges are preferred, it is on account of their greater cheapness. Sometimes the hole in the stones by which they are hung is round, sometimes it is square. The Commissioners think the round hole less liable to cause cracks.

In Sheffield workshops it was found that there was often a gangway or passage in front of the stone, and that it was placed in front of a fireplace. Men had several times been injured either through chancing to be passing along the gangway when a stone flew, or through having congregated round the fire either to warm themselves after having been engaged in a sedentary occupation, at which they can hardly help getting splashed, or to dry their work. The running of a stone "before a fireplace" is forbidden; but as the Factory Act of 1895, in which this prohibition occurs, did not specify the space that was to intervene between the stone and the fire, it has proved ineffective. In Birmingham this danger is avoided. There is a thick wall in front of the workman, and the stoves for heating and drying are behind and out of the range of flying stones. The Commissioners advise that guards should be put over all stones, and that before use they should be tested by "racing," i.e., running at a higher rate of speed than will be expected of them regularly, this test to be carried out in a machine where there would be no danger to anyone if they should fly.

